# When the Internet Gets Under Our Skin: Reassessing Consumer Law and Policy in a Society of Cyborgs

**DOI:** 10.1007/s10603-024-09581-y

**Published:** 2025-01-23

**Authors:** Benjamin Clubbs Coldron, Guido Noto La Diega, Christian Twigg-Flesner, Christoph Busch, Tabea Stolte, Marc-Oliver de Vries

**Affiliations:** 1 University of Stirling, Stirling, Scotland; 2 University of Warwick, Coventry, England; 3 Universität Osnabrück, Osnabrück, Germany

**Keywords:** Internet of Things (IoT), Privacy, Security, Data, Identity, Human adaptation

## Abstract

In this article, the authors identify and explore the phenomenon of consumer cyborgification and ask what the legal and ethical implications of this emerging trend are. They consider whether fundamental legal principles, concepts, and assumptions in various EU acts and directives are adequate to address these challenges or whether these need to be reassessed in light of novel forms of vulnerability. They also ask what alternatives might be suggested. In the era of the consumer Internet of Things (IoT), consumer expectations of privacy, security, and durability are changing. While the consumer uses of the IoT often revolve around improving efficiency (e.g., of the body, the home, the car) and enhancing experiences through datafication of our bodies and environments and personalization of services and interfaces, the power of IoT companies to influence consumer behaviours and preferences is increasing in part because the hybridization of humans and machines. Cyborgification allows our behaviours to be individually and continuously monitored and nudged in real time. Our bodies and minds are reflected back at us through data, shaping the narratives we tell about ourselves and our surroundings, and this is creating new lifeworlds and shaping our preferences, roles, and identities. This presents novel benefits, as well as risks in the potential exploitation of novel vulnerabilities. With technology under the skin, both metaphorically (in relation to products that become a sensory accessory to the body and influence the perception and physical reality of one’s body and lifeworld) and literally (in the form of microchips, cybernetic implants, and biometric sensors and actuators), cyborg consumers are more vulnerable to manipulative practices, unfair contractual terms, automated decision-making, and to privacy and security breaches. Cyborg consumers are therefore more susceptible to damage, financial and physical, caused by defective products, low-quality services, and lax cybersecurity. Law, policy, and practice must go further than merely enhancing transparency and consent processes and prohibit practices and business models that are premised on manipulating the need to anticipate and manage the working of technologies under the skin, i.e., that which undermines consumer and public interests systematically. The law needs to be agile and responsive to the changes the IoT has established in the consumer–producer relationship. Consumer laws, including the contractual/consenting process itself, must be reviewed and reimagined to ensure more robust protections.

## Introduction

The consumer Internet of Things (henceforth “IoT”), refers to the network of everyday physical devices (“Things”) that are embedded with sensors, software, and connectivity capabilities, allowing them to collect and exchange data with other devices and systems online. These devices are designed for the consumer market and include smart home devices such as smart thermostats, voice-controlled smart speakers, smart appliances (e.g., refrigerators, washing machines), and home security cameras; wearables such as fitness trackers and watches; and connected cars.

The consumer IoT has become increasingly popular, offering new ways to interact with and manage our environments (Sinha, 2023). However, as we become increasingly reliant on IoT technologies, and they are integrated under the skin in the form of implants and into our lifeworlds through datafication and personalization, we must also be aware of the potential risks. The combination of increased dependency and presence in our lives of insecure products with actuation capabilities providing greater opportunities for companies to engage in unfair commercial practices and enforcement of potentially unfair contractual terms, we argue, raises novel challenges for consumer protection and rights.

While others have already shed light on human vulnerability caused by machines (Oudshoorn 2015) and machine vulnerability caused by humans (Alsharif et al., 2022), the contribution of this paper is to identify that as connected and remotely controllable technology goes under our skin, this cyborgification creates a human–machine symbiosis in which human capabilities are enhanced (Inga et al., 2023), but that increase and complicate our vulnerabilities. Anticipating and taming Things under the skin constitute a new form of invisible labour, and companies have novel capabilities to manage what might be termed “consumer-employee” behaviours (Oudshoorn 2015). As a response, the legal conceptualization of vulnerability must become more contextual, granular, and personalized. Cyborgification creates new types of vulnerability “as an internal rather than an external threat and as harm you may try to anticipate but can never escape” (Oudshoorn 2015, p. 267). Our concept of cyborg vulnerability builds on the literature on digital vulnerability (Helberger et al., 2021a) and is characterized as a relatively new development in the gradual, longstanding, and ongoing process of human–machine hybridization (Totschnig, 2022).

Cyborg vulnerability is relational, mutually reinforcing, and is not restricted to those with inherent or socio-economic, physiological, or psychosocial disadvantages. It encompasses the dependence consumers experience, on those providing updates cloud and edge computing and general product support, as well as the intimacy, proximity, and constancy of the Things that we integrate into ourselves from insulin pumps and glucose monitors to bio-hacking CT scanning headphones.

Cyborg consumers are more susceptible to data breaches, surveillance, and manipulation through targeted advertising and nudges as well as new forms of unfair commercial practices and contractual terms enforcement (e.g., through bricking). Of course, cyborg manipulation is already regulated to some extent. For example, under Article 22 of the General Data Protection Regulation (GDPR), data subjects have the right not to be subject to a decision based solely on automated processing, including profiling. One concrete example of efforts to tackle the gap between law and practice is in the new approach to technical standards exemplified by the AI Act whereby European Union (EU) binding legal requirements will be complemented by voluntary harmonized technical standards developed by the European Standardisation Organisations (Micklitz, 2023), and this could be a fruitful line of inquiry.

In addition, the Digital Services Act Article 27 embraces a legal design approach which seeks to oblige companies in the design stage of product development to think about measures to enhance transparency and accountability in relation to recommender algorithms utilized by providers of online platforms. More crucially, however, by requiring measures to allow users to modify the parameters related to recommendations and personalized content, cyborg consumers might be empowered to control the information they receive, which is essential for maintaining autonomy in a highly personalized and data-driven environment (Micklitz, et al., 2024).

The implementation of these and various other consumer rights and protections under EU law, in the design and maintenance of cyborg technologies, is complex and requires a more focused empirical study of the ways abstract legal requirements translate into the practical consumer experience. This paper is a tentative exploration of some of these issues drawing on empirical work.

## Methodology

A pragmatic mixed methodology is used whereby, to answer legal research questions around what the law is and how it is interpreted, we use doctrinal methods, and to answer empirical questions around IoT company culture and practices, consumer experiences, and behaviours, we use qualitative methods including interviews and focus groups with both consumers and IoT experts in the industry.

The empirical investigation employs a multi-faceted approach, focusing on consumer issues within the connected car, wearables, and smart home IoT sectors. The organizational sample includes both large and atypical companies within these sectors.

Interviews with people working in IoT companies were conducted to gain insights into various aspects, such as legal obligations, contracting, dispute resolution, and consumer support. We secured three interviews with connected car industry experts, who were also consumers of the product, and one interview with a smart home industry expert. We conducted two focus groups with smart watch consumers in Scotland and Germany with a total of 12 participants. The focus groups aimed to explore consumer expectations of companies when IoT products/services encounter issues. Interview techniques included face-to-face recordings, online video calls, and phone calls, with a semi-structured approach guided by predetermined topics. Textual data analysed included reports and surveys from consumer rights organizations, blog posts, and reviews related to IoT products and services, privacy notices, terms of use, sale and subscription contracts, licence agreements, and warranties.

Clarke and Braun (2017) thematic analysis was utilized, yielding insights into the legal and practical challenges faced by consumers, how they experience the consumer/producer interaction, and potential solutions to the issue of cyborg vulnerability. This integrated design ensures a robust exploration of various consumer issues within the evolving landscape of IoT.

### The Notion of the Cyborg

The term cyborg, short for cybernetic organism, was first coined by the biophysicist Manfred Clynes and the neuroscientist Nathan Kline in 1960 to describe a hypothetical human–machine hybrid (Clynes & Kline, 1960). Their research explored how integrated mechanical components might support “the task of adapting [one’s] body to any environment [one]… might choose.” Since then, the concept of the cyborg has been explored in the fields of sociology, philosophy, and cultural studies, among others.

In consumer law, some notable contributions include Wittes and Chong (2014) who have considered the “always-on” aspect of certain intimate technologies, embedded or not, and suggested novel approaches to ownership rights, data rights, and security needs may be required for what they call cyborg consumers. Chris Habels Gray (2001, 2018) has also written extensively on the ethics and politics of cyborgification in the conflict between intellectual property rights and consumer transparency rights and concluded that existing laws and statutes are less than fully adequate to regulate and guide the development of cyborg technologies. He has proposed a cyborg “Bill of Rights” which includes the right to freedom of consciousness and freedom of information, arguing that institutional and corporate use of information to coerce or otherwise unduly manipulate or act upon citizens should be prohibited (2001, pp. 27–29). (Table 1)

**Table 1:** Organizational sample for analysis of legal documents

Use case	IoT companies
Connected cars	Mercedes; BMW; Audi; Tesla; Ford; General Motors; Toyota; Honda; Hyundai; Volvo; Renault; ONTO
Wearables	Tracktive; Fitbit; Apple; Garmin; Samsung; Fossil; Jawbone; Misfit; Withings; Polar; Suunto; Dexcom; Abbot
Smart home	Amazon; Google; Apple; Hive; Samsung; TP-Link; Netatmo; Philips; Nest; Honeywell, North Tech, Albyn Housing

Current corporate practice in the IoT sector, particularly with intrusive semi-permanent or permanent Things placed on or in the body (i.e., cyborg technology “under the skin”), arguably occupies a grey area between illegal and legal manipulation. The fine line between personalization and undue influence amounting to unfair commercial practices under Article 6 of the Unfair Commercial Practices Directive (UCPD) is a case in point. Given the limited interfaces by which consumers interact with cyborg technologies devices (e.g., an implant may be only modifiable through a specific app or may even be restricted to the company supporting it, a hearing aid or smart headphones may rely on audio to convey information), communicating all relevant terms and conditions and privacy policies becomes difficult and it is inevitably curated. Personalization through Things allows the choice architecture presented to consumers to be adapted in real time and on an individualized basis. The omission of information about how personalization is utilized or the impact of personalization can have on consumer autonomy and choice is arguably a problematic omission under Article 7 of the Unfair Commercial Practices Directive (UCPD) and enhancing and properly enforcing transparency and control rights over the parameters of recommendation algorithms as in Article 22 of GDPR appears necessary.

The vague ways in which personalization is presented in privacy policies may create uncertainty for consumers, raising ethical concerns about transparency and disclosure. The fact that data is so granular and IoT devices so ubiquitous may enable companies to exert psychological pressure at precisely targeted moments, exploiting vulnerable emotional states to influence transactional decisions, roles, and preferences. With the invention of ever more intrusive sensors, placed permanently or semi-permanently on or in the body (see Apple’s patent for electroencephalography (EEG) headphones) and AI-powered analytics creating inferences and predictions combined with producers’ direct remote control over devices, intimately connected with, or integrated into, a person’s body the potential for unfair commercial practices increase. A fundamental question arises with these often-benevolent technologies—when does influence become unfair? How do we distinguish between the use of IoT devices to coerce or unduly influence consumers and the influence which is expected and desired?

The influence of devices over our bodies, minds, and behaviours is all the more effective if Things are under our skin in the sense that they are embedded into our bodies (like glucose monitors or hearing aid implants) and our identities (as with a smartwatch becoming a fundamental aspect of everyday life and an identity signifier as discussed in one of our interviews—not all smartwatch users will become cyborgs) and connect with things hidden in the everyday environment. This raises the question of what forms of manipulation should be permissible in consumer law, whether or not people are informed and consenting, and which types of actors might legitimately engage in such conduct, where the line that is drawn is currently ambiguous.

Part of the reason for such ambiguity is that the cyborg represents a new form of identity that challenges traditional categories of gender, race, and class and opens new possibilities for social transformation, negative and positive (Haraway, 1991). Becoming entangled in such relationships with technology leads to a change in people’s lifeworld (i.e., the horizon of all our experiences and the background on which all things appear as themselves and meaningful) and identities, and this in turn creates a feedback loop where technology and the way it is designed to respond to these modified behaviours and preferences. These have profound effects on the way that governmentality and other technologies of power (e.g., disciplinary techniques, surveillance systems, and biopolitics) operate (Foucault 1980).

At present, many of these technologies are controlled by companies and are utilized to increase profit. Cyborg bodies and minds can be adapted and modified to suit the environment of surveillance capitalism (Zuboff, 2019). Zuboff’s thesis contends that tech companies, as surveillance capitalists, exploit advanced digital technologies to extract, analyse, and commodify extensive personal data, and this fundamentally reshapes power dynamics, eroding privacy and posing challenges to democratic norms. Vast quantities of information are gathered and assessed, and the outcomes of various algorithms shape our digital, social, and physical environments. Our responses to these customized experiences generate additional data, which is then collected and analysed in an ongoing cycle. With the growth in the size and complexity of deep-learning AI, learning, reflecting, and then shaping our preferences and monitoring our responses which are then fed back into the machine learning system, this trend appears to be accelerating.

Placing remotely controllable technologies under our skin can be both empowering and disempowering simultaneously. The problem that consumer law and policy must confront is that the IoT is a powerful tool which, in the hands of unscrupulous businesses, can and will be used in ways that are contrary to the public interest be it public health, reducing waste and pollution, undermining security, or distorting markets. Re-asserting vulnerability and consumer welfare, as an essential ethical underpinning for consumer protections in law and policy, provides an intellectual, moral, and epistemological justification and basis for smarter consumer laws.

According to Ugo Mattei (2022), the constant connection between individuals and Things in the IoT has significant socio-anthropological implications. Connection transforms citizen-consumers into cybernetic organisms consisting of the physical person and the hardware and software which either physically or metaphorically gets under the skin. This not only involves implants and connected technologies placed on or in the body, but also technologies which serve as additional sensory organs which influence how we see ourselves and the world around us. This leads to the emergence of new categories of personhood (Hacking, 1995). The modern cyborg perceives of and acts in the world in a different way as a result of their engagement with technologies which augment capabilities and direct experiences and generate data which then informs the perception of oneself and the world around (see, for example, Baltzer et al. (2014), Berberian (2019), Berberian et al. (2012), Bütepage and Kragic (2017), and Inga et al. (2023)).

In the age of the cyborg, humans and computers are increasingly converging on an individual as well as on a societal scale. Governmental, corporate, and individual decision-making is increasingly modified, influenced, and in some cases determined using data gathered from multifarious sensors (Fleischmann, 2014). For cyborgs, decisions made can be implemented on their bodies remotely. This creates novel forms of consumer vulnerability because although the data are gifted back to us in accessible ways and outsourcing certain decisions can be convenient, these gifts create dependencies which are complex and multifaceted (Russell, 2021).

Drawing on research conducted among technology developers and marketers of personal health technology, Schüll (2018) considers how IoT companies “design self-care” into their products in the form of motivational feedback loops and “micronudges” that operantly condition behaviour. Normative social expectations of health, beauty, and success are reflected and reinforced in target numbers, scores, and gamified incentives (Depper & Howe, 2017). These systems can shape consumers’ interest, preferences, and identities. A “numerical ontology” comes to transform our bodies into a means to an end. Our minds are cast in the role of engineers forever seeking to make our physical apparatus more efficient. Through repetitive practice, these roles are suffused with our very identities and the way we relate to ourselves (Butler, 1993; Oxlund, 2012).

Smith and Vonthethoff warn that “bodily intuition is being outsourced to, if not displaced by, the medium of unbodied data” (2017). Consumers subject to such abstraction can experience anxiety, a loss of autonomy, and addiction (Lomborg et al., 2018; Pink et al., 2018; Schull 2018). Innovative technologies are therefore producing new ways of experiencing the world as our digital and physical selves are split and then remoulded. This was reflected in our empirical work on smart wearables where some participants suggested that numerical targets in the data gathered about themselves through Things became a powerful lens through which they perceived themselves and their actions. Here is one illustrative example:

It tells me how many steps I did today. It tells me whether my heart rate was high, so maybe I was anxious that day, or it was a stressful day… it tells me how many calories [I’ve] burned… I like data I’m a quantitative scientist… I look at the graph where… it shows my hard drive over a few days. (Participant 6, Wearables Focus Group 2)

Through IoT technology, we cannot perceive or understand our experiences and emotions other than as objectivized data. Human behaviour is becoming more predictable because our behaviours and preferences are perceived and managed through algorithmic regulation (Russell, 2021). We are adapting to the machines rather than they adapting to us. Finding new ways to support consumer (or human) agency in these large, hybrid networks is an important challenge (Fenwick & Jurcys, 2022).

Having described the notion of the cyborg, the technology we associate with this concept (i.e., technology under the skin both metaphorically in relation to products that influence the perception of one’s own body and environment and literally) and some of the general consequences of cyborgification in changing consumer behaviours, preferences, identities, and lifeworld’s, we now move on to discuss the specific consumer vulnerabilities that result. In the next section, we describe vulnerability as a general concept and then look at cyborg vulnerability as a sub-category.

### Cyborg Vulnerability

From the literature, we can identify three broad categories of vulnerability:

1. Inherent (or pathogenic) vulnerability—vulnerabilities that arise from inherent characteristics of the individual such as age or disability (Havrilla, 2017)
2. Contextual (or relational) vulnerability—vulnerability caused by reduced capacity, power, or control to protect one’s interests relative to other agents (Helberger et al., 2021a, 2021b)
3. Universal (or ontological) vulnerability—human bodies are fragile and neither independent nor self-sufficient. We are all vulnerable (Fineman, 2008)

Consumer vulnerability has been theorized in several ways which seek to integrate some of the definitions above. Lim and Letkiewicz (2023), for example, argue that inherent (the “disadvantaged consumer framework”) and relational vulnerabilities (the “vulnerable consumer framework”) are both useful in identifying potential risks and consumer harms (this is a development of the work of Baker et al. (2005), Commuri and Ekici (2008), Garrett and Toumanoff (2010), and Raval (2020)). Frameworks focusing on “disadvantaged” consumers have previously been utilized to identify the consumers most likely to experience vulnerability in the marketplace. This was based on the idea that certain demographic characteristics, such as low income, less education, minority status, and old age, render a consumer disadvantaged (Garrett & Toumanoff, 2010; Lim & Letkiewicz, 2023).

This is supplemented by the idea of relational vulnerability which is “a state of powerlessness that arises from an imbalance in marketplace interactions or from the consumption of marketing messages and products” (Baker et al., 2005, p. 34). Although consumers in some categories are seen as permanently vulnerable because of certain characteristics, it is perhaps more accurate to say that a universal potential for vulnerability is activated when a person is placed in a situation where another agent has a degree of power and control over them (which inherent vulnerabilities may contribute to). In this framework, vulnerability arises from multiple factors including individual characteristics (e.g., cognitive capacity), individual states (e.g., life transitions), and external conditions (e.g., discrimination).

Domurath (2018) similarly considers vulnerability to be a complex interaction between inherent and situational vulnerabilities. She argues that consumer vulnerability arises from structural imbalances in information, power, and expertise between consumers and financial institutions in mortgage markets. The goal of addressing consumer vulnerabilities, according to Domurath, is to enhance consumer welfare and not simply to level the playing field. This approach underscores the importance of developing universal consumer-centric policies and regulations that prioritize transparency, fairness, and the protection of personal autonomy.

Although these various theorizations of consumer vulnerability are nuanced and pragmatic, translation of these complex concepts into law, policy, and practice has not always followed suit. Academics such as Fineman argue that judges in applying consumer law still subscribe to the foundational myth of the autonomous, rational subject (Fineman, 2008; Helberger et al., 2021a, 2021b) which makes the presumption that, in general, consumers are the opposite of vulnerable. Of course, it might be countered that the very existence of the consumer law regime is a tacit acceptance of the vulnerability of consumers vis-à-vis producers. The argument is perhaps more about how we determine the level or type of vulnerabilities where legal rights and protections are justified. The question is who is deserving of protection, and this has historically been viewed as a determination of whether a consumer is guilty of culpable irrationality (because of laziness or ignorance) or a victim of blameless irrationality (as a result of cognitive impairment).

In the age of datafication and cyborgification, the assumption that any consumer might act in rational ways cannot be sustained (Esposito, 2017, 2018). Our ability to make rational, autonomous decisions is influenced by numerous factors, including digital architectures (Helberger et al., 2021a, 2021b), information overload (Hu et al., 2023), and individual digital literacy (Van Ooijen & Vrabec, 2019). Indeed, for cyborgs dependent on companies to maintain and support Things intimately connected to them, it may well be rational to avoid any confrontation or conflict caused by seeking to assert consumer rights, or it may well be made hugely inconvenient and time-consuming. Establishing rationality appears to be a rather subjective or ideological exercise.

When looking at the behaviour of consumers, it is clear we all have “dispositional” vulnerabilities. However, these are latent and are activated only when we interact with agents powerful enough to exploit those underlying frailties. Successfully exploiting vulnerabilities that arise in such an interaction converts dispositional vulnerability into “occurrent” vulnerabilities (Helberger et al., 2021a, 2021b). Cyborgification means that dispositional vulnerabilities can be detected in real time and exploited more readily because Things are always on and follow us into even our most intimate environments. This means that “cyborg vulnerabilities” lie somewhere between universal and relational vulnerability.

Cartwright (2015) applying a relational concept of vulnerability to financial markets identified several characteristics of the interaction, context, and environment that converted potential to occurrent vulnerabilities. These included information vulnerability (linked to the ability to obtain and understand information or to make the right choice), pressure vulnerability (greater liability to hard pressure selling techniques), supply vulnerability (inability to afford essential goods or services or having less choice within an affordable price range), redress vulnerability (difficulty in seeking remedies for wrongs suffered) and impact vulnerability (being more affected by making bad choices).

These categorizations allow us to determine more confidently and precisely the obligations that companies might have towards consumers of products or services in different situations in consumer law. If there are broad similarities in the way that vulnerabilities are manufactured and exploited across different sectors, then a set of general rules and regulations (that apply to, but also beyond, the IoT) might be appropriate. However, we may also discover types of cyborg consumer/company interactions, characteristics, or outcomes, which demand specific legal interventions. For example, where medical devices are concerned defective products or unfair commercial practices carry significant and particular risks, user protections look very different from domestic appliances or smart watches. Whether cyborg vulnerabilities need to be dealt with specifically or a more general approach to digital vulnerabilities might be sufficient is up for debate.

Cyborg vulnerability and digital vulnerability—a concept developed by Helberger et al. (2021a)—are distinct but related. Helberger and her colleague’s concept of digital vulnerability addresses the various susceptibilities that individuals face in the digital environment, encompassing personal, situational, and systemic factors. On a personal level, digital vulnerability can be significantly influenced by technical literacy, psychological state, and age or experience. Individuals with limited knowledge and skills in using digital tools are more prone to misunderstandings and exploitation. Mental health conditions, stress, and cognitive overload further impair the ability to make informed decisions online. Older adults and those less familiar with digital technologies often encounter greater challenges in adapting to new digital environments.

Situational factors also play a crucial role in digital vulnerability. The availability and quality of internet access, digital devices, and technical support can greatly impact a person’s digital experience. The context in which digital technology is used, such as public versus private spaces, can influence the level of risk; for instance, using public Wi-Fi may expose users to higher security risks. Systemic factors include aggressive market practices such as targeted advertising and personalized content that allow companies to exploit vulnerabilities by manipulating consumer preferences and behaviours (Zuboff, 2019). There is evidence that consumers adapt and resist these strategies, but business practices also evolve in a predator–prey dynamic (Acquisti et al., 2016; Calo 2013; Erevelles et al. 2016).

Helberger’s concept emphasizes the need for a holistic approach to understanding and addressing the challenges individuals face in the digital world, but emphasis is also placed on solutions in the digital sphere. If clear and accessible information, as well as robust and ongoing cybersecurity updates, is provided, then digital vulnerabilities may remain latent. By considering personal, situational, and systemic factors, policymakers, businesses, and technologists might better protect and empower users, ensuring a more inclusive and equitable digital environment. It is our view that in the context of cyborg technologies, in the form of IoT consumer products that reach “under the skin,” this concept does not provide adequate focus on the way in which physical products with digital services can make these issues even more acute and introduce novel problems.

Cyborg vulnerability (though including a digital vulnerability in relation to the IoT apps, interfaces, and software and following the holistic approach) is slightly broader than digital vulnerability in the sense that Things create novel vulnerabilities in the collision of physical/digital spheres (Haddow, 2021; Oudshoorn 2015). Digital vulnerability emphasizes (though is not restricted to) the ways in which vulnerabilities can be exploited through designing software and algorithms to shape and personalize digital environments. Cyborg vulnerability, while encompassing this concept in relation to the digital aspects of physical products that modify the human body or experience, also identifies vulnerabilities connected to the physical actuation capabilities of products with digital elements and the business models dependent on datafication. When the internet becomes physical, the ontological and epistemological consequences are unpredictable.

“Cyborg vulnerability” refers to the unique vulnerabilities that arise from the integration of human beings with technology, blurring the boundaries between humans and machines. The concept refers not only to the way that the digital environment, in interaction with human frailty, allows opportunities for financial exploitation and attention capture but also to the way in which new types of personhood, coupled with remote-control capabilities and dependencies, are cultivated by companies. Solutions are indicated in regulating software and hardware design, reassessing contracting processes and fundamental legal assumptions, and creating more opportunities for consumer influence on technical standardization (Micklitz, 2023). Huge public interest questions also arise in relation to how cyborg technologies can be used to support public health, reduce environmental impacts, and optimize productive and creative capacities but also to the profound negative consequences this can have for individuals.

One essential characteristic of cyborg vulnerability is its deeply personal nature of integration of digital and biological systems. Unlike digital vulnerabilities associated with abstract data or systems, cyborg vulnerabilities directly impact individuals’ physical and cognitive functions. Examples include smart glucose monitor implants, microchip car keys implanted into people’s hands, or even ear pods that conduct EEG, EMG (electromyography), track eye movement, and monitor ECG (electrocardiography). These devices are monitoring not only external behaviours but also internal brain processes potentially giving companies the ability to monitor and perhaps soon influence behaviour in more effective ways. Malfunctions or exploitation of cybernetic implants or enhancements can lead to bodily harm, loss of autonomy, or even existential threats. They are blurring the boundaries between medical and consumer devices.

Moreover, cyborg vulnerability introduces new challenges to traditional notions of privacy, consent, and agency. The data generated by bodily enhancements or implants, such as biometric information relating to ovulation, neural activity, and gut health, raise concerns about surveillance, identity theft, and unauthorized access. Individuals may face coercion or manipulation through their cybernetic interfaces, undermining their ability to make autonomous decisions. Cybersecurity becomes much more direct in the risks being physical as well as abstract but also in the way that biometric data might be used and appropriated. The sensitivity and granularity of this data significantly amplify the potential for misuse and exploitation, thereby increasing vulnerability.

Cyborg vulnerability can be seen as a subcategory of digital vulnerability with the use cases providing added layers of complexity and depth. While digital vulnerability deals with risks associated with data privacy, security breaches, and exploitation of digital behaviour, cyborg vulnerability extends to the manipulation of people based on indirect and potentially direct power over bodily functions and mental states. This means that law must not only remain abstract, but also influence the physical design of such IoT products—Law by Design becomes paramount and interdisciplinary influence over technical standardization processes urgent. Interdisciplinary approaches, combining insights from bioethics, cybersecurity, physiology, and psychology, are necessary to understand the full spectrum of risks (Ducato et al., 2024). Continuous monitoring and analysis of both digital and physiological data may be required to detect anomalies and potential vulnerabilities to cybersecurity breaches.

Representatives of large organizations (not just private companies but also healthcare providers, local authorities, and more) in creating databases and profiles can fall into a habit of reducing cyborgs to Things (i.e., data producers, sensors, and actuators) rather than recognizing them as citizens with rights (as means rather than ends). An objectifying discourse develops through processes of quantification and abstraction and can undermine consumer trust and confidence that their privacy and autonomy are given due recognition. Frischmann and Selinger go as far as to say we are reduced to “cogs in the machine of our own lives” (2018) and increasingly passive, non-agential, irresponsible, and ignorant. “Human–robot interaction research” depicts the human within the IoT ecosystem (and particularly the relationship between consumers and Things) in mechanistic and potentially reductive or deterministic ways (Cross & Ramsey, 2021; Sequeiros et al., 2022). In the business discourse relating to the consumer IoT, a user’s role is often bracketed out of the IoT ecosystem as a non-agent (Lee, 2019; Sestino et al., 2020).

Cyborgs can also become “de-sensitized” to their own bodies through overreliance on IoT devices to measure and interpret bodily changes. IoT companies design choice architectures and feedback loops into IoT systems and manufacture “micronudges” that operantly condition behaviour in ways that undermine consumer choice and push them into particular habits (Schull, 2016). Psychological pressure, based on data that personalize consumer experiences, is used to incentivize certain types of consumption and even change preferences (Depper & Howe, 2017). Business in the IoT sector can therefore create cycles of dependency as a “numerical ontology” transforms our bodies into measurable units. Our minds are cast in the role of engineers forever seeking to optimize and make the machine more efficient. We play these roles enough that they become suffused with our very identities and the way we relate to ourselves and the world around us (Oxlund, 2012).

Consumers’ sensory experiences are therefore being outsourced to, if not displaced by, data (Smith & Vonthethoff, 2017). New technologies are producing new ways of experiencing the world and potentially rewriting our very souls (Hacking, 1995). The proliferation of networked physical products and devices, implanted with sensors, software, and other technologies, and the integration of these devices into our environments and our bodies create new lifeworlds. In the IoT, these lifeworlds can be personalized to each individual and so the experience is atomized; this undermines consumer organization or even the possibility of collective experience that can be the basis of arguments and campaigns for change.

In practice, it appears that the IoT renders cyborgs more “programable” and thus vulnerable to being used as means rather than ends whether this use is beneficent or otherwise. By engaging in various online activities, consumers generate a substantial amount of personal data that companies collect and process (Acquisti et al., 2015). This data is then used to customize IoT services, providing personalized communication and actions tailored to individuals’ characteristics, interests, and behaviours (Bol et al., 2018). In “smart” environments, personalized marketing communication plays a crucial role in shaping user experiences (Strycharz & Duivenvoorde, 2021). Consumer profiles are based on various inferences made from the combination of data points which, in isolation, may be seen as relatively mundane.

The ways in which cyborgs are rendered vulnerable can be described in stages. Firstly, engagement with digital services and platforms results in the accumulation of large databases made up of individual consumer profiles which purport to outline the consumers’ preferences and consumption habits in ways that allow prediction of future behaviours and preferences. IoT companies then feed data into these databases using devices which follow the person around in their everyday life (on/in their person, in their home, and in their vehicles) and gather real-time, detailed data. Cyborgs have more attack surfaces for hackers, marketers, and influencers to exploit and also greater capabilities to nudge behaviours or change environments through IoT actuators. As these attack surfaces are tested, the reaction of consumers to various nudges or marketing strategies can be monitored, and the strategies refined over time. Finally, these more sophisticated and detailed consumer profiles can then be sold to consumers, companies, or public authorities in the form of services which modify and shape people’s preferences and behaviours further. In the next section, we move on to discuss how the law deals with this.

### Cyborg Law and Policy

For a broad overview of cyborg law, scholars such as Viljanen (2017) have discussed the issue of regulating cyborg societies arguing that a new kind of modality of power is emerging. Regulation, they argue, now involves manipulating information inputs, intervening in the “material constitution of the cyborg cognitions” through the construction of “socio-technical assemblages that constitute the cyborg agents” cognitions or determine their behaviour (Viljanen 2017 pp. 292–1300). This effectively amounts to “psycho-morphing” humans to make them more useful components in the cyborg system (Viljanen 2017 pp. 1300–4). This approach treats cyborgs as Things in the system rather than autonomous agents. From the perspective of consumer protection, the realization of such technologies of power—in the Foucauldian sense (see Nilson, 1998)—would present a major risk to consumer autonomy, privacy, and security.

Quigley and Ayihongbe (2018) discuss product liability, data, privacy, cybersecurity, and intellectual property rights and conclude that the law currently provides inadequate answers to many issues raised by cyborgification. They argue that satisfactory consumer protection requires adapting the conceptual and philosophical underpinnings of the law, as well as the law itself. Vulnerability is one such underlying principle of consumer law (i.e., consumers are vulnerable vis-à-vis producers and so require bespoke protections) and the concept of vulnerability is often interpreted restrictively.

In addition, Karampatzos (2020), Ben-Shahar and Porat (2021), and Busch and De Franceschi (2020) have considered algorithmic personalization and its impacts on consumer law policy and algorithmic regulation. Their works highlight how the integration of IoT devices into our bodies can create new vulnerabilities, as these devices collect and process vast amounts of personal data to tailor experiences and services to individual preferences. This personalization can subtly influence consumer behaviour and decision-making processes, raising significant concerns about autonomy and privacy.

Karampatzos (2020) discusses how private law can incorporate behavioural economic insights to address these challenges, suggesting that nudging can be used to guide consumer behaviour in more transparent and beneficial ways. Ben-Shahar and Porat (2021) delve into the concept of personalized law, proposing legal frameworks that adapt to the unique characteristics and behaviours of individuals. This approach, while potentially increasing efficiency and fairness, also requires robust safeguards to protect against the misuse of personal data and manipulation.

Busch and De Franceschi (2020) explore the regulatory implications of algorithmic personalization, emphasizing the need for policies that ensure accountability and fairness in the deployment of personalized algorithms. They argue that algorithmic regulation must balance the benefits of personalization with the protection of individual rights, particularly in the context of invasive IoT devices that blur the boundaries between humans and machines. The convergence of these perspectives underscores the necessity of rethinking legal and regulatory frameworks to address the unique vulnerabilities posed by cyborgification, ensuring that technological advancements enhance rather than undermine consumer autonomy and privacy.

Emphasizing such autonomy, Ugo describes cyborgs as consumer-citizens and contrasts them with “citizen-consumers” of the past whose activism he argues created a hard limit to capitalist extraction. Accordingly, “being a cyborg is a social relationship not only with the screen to which we are glued, but also with tens of billions of other Things that populate the IoT” (Ugo, 2022, p. 615). Cyborgification promises consumers the ability to adapt to an increasingly complex world in which the digital and the physical realms overlap and our bodies and minds connect and share information with Things autonomously (or unconsciously). Things perform specific tasks such as planning exercise routines, completing domestic chores, or driving safely and efficiently. These tasks are at present performed in interaction with the individual and the technology with some aspects automated and some aspects human-led. The performance of these tasks can create an experience of increased agency and competency, dependent on human–machine symbiosis (Inga et al., 2023).

The law, in the EU and in other jurisdictions such as England and Scotland, has been updated periodically to deal with digital vulnerabilities (see, e.g., in the EU, the Digital Services Act, Digital Markets Act, the AI Act, the proposed AI Liability Directive; and in the UK, the Online Safety Act 2023, the Digital Markets, Competition and Consumers Bill, the UK Product Safety Review, and the AI whitepaper from the Department for Science, Innovation and Technology, UK Government, 2023). The pace of change is such that legislators must continuously reassess and update our system of rights and obligations in order that novel vulnerabilities and power imbalances are taken into consideration as technology and business models develop.

As we have seen, vulnerability is often conceptualized as an inherent characteristic of the individual. Contextual and relational factors are certainly considered in law, though often as an afterthought. In this section, we look in turn at unfair commercial practices, unfair contractual terms, platform regulation, cybersecurity, data protection, privacy, and artificial intelligence.

### Unfair Commercial Practices

The UCPD pursues three main objectives: harmonizing EU law, safeguarding consumer economic interests, and establishing a level playing field for businesses across the EU. Prohibited practices under the UCPD include those infringing professional diligence and misleading or aggressive commercial practices that distort the transactional decisions of the average consumer. The directive employs a two-part test to assess unfair practices based on professional diligence requirements and the material distortion of the average consumer’s economic behaviour. Misleading practices involve false information or omissions leading to decisions the average consumer would not have otherwise made. Aggressive practices undermine consumer choice through harassment or coercion.

The UCPD requires additional protection for consumers who are “particularly vulnerable due to their mental or physical infirmity, age or credulity” (UCPD, Article 5.3). According to recent guidance, mental and physical infirmities are inherent characteristics, but credulity can in practice be manufactured by circumstance through dark patterns, restriction of information, pressure selling, and other forms of manipulation embedded into IoT architectures (European Commission, 2022a, 2022b).

Article 7 of UCPD Part 4a (introduced by Directive (EU) 2019/2161) creates transparency requirements in online marketplaces to ensure that consumers are provided with clear and relevant information about their rights and the obligations of sellers when engaging in online transactions. It requires online marketplaces to inform consumers about the key parameters determining the ranking of offers presented to the consumer because of a search query. It includes the necessity to provide information about the main parameters and their relative importance.

To discuss the vulnerable consumer standard in the UCPD, it is important to understand the broad purpose of the directive and how the vulnerability concept is operationalized. Article 5 of the UCPD (the general clause) prohibits unfair commercial practices that distort the economic behaviour of the average consumer. For a practice to be characterized as unfair, for example, it might contravene the requirements of professional diligence and materially distort the economic behaviour of the average consumer.

To decide whether a commercial practice is unfair, it needs to be assessed against one of the consumer standards, namely the average consumer or the vulnerable consumer. The average consumer is the benchmark of the UCPD, defined as a “reasonably well-informed, reasonably observant and circumspect” individual (Court of Justice of the European Union (CJEU) 16.07.1998 Case C-210/96 (Gut Springenheide) ECLI:EU:C:1998:369; Esposito & Grochowski, 2022). This reflects assumptions considered above about the rational and informed consumer which have come under sustained criticism. However, for instances where a product is harmful or particularly harmful to vulnerable consumers, the “average vulnerable” standard is employed. If evidence is raised that a company is utilizing consumer data from cyborg technologies for commercial gain (e.g., withholding updates so the consumer purchases a new product), the court might consider how this practice would impact the average person with diabetes mellitus rather than how it impacted on a particular person (e.g., the company may use profiling to identify that this consumer was pregnant and therefore more susceptible to the marketing pitch). It is worth noting that between the average and vulnerable consumer, there is the uniformed, unobservant, and/or careless consumer who will not be shielded from the effects of their own incompetence.

Article 5.3 of the UCPD states that commercial practices that are likely to materially distort the economic behaviour of a clearly identifiable group of consumers who are particularly vulnerable to the practice or the underlying product because of their mental or physical infirmity, age, or credulity shall be assessed from the perspective of the average member of that group. There is a wealth of literature considering the implications of this provision (see, for instance, Friant-Perrot (2016), Galli (2022), Hacker (2023)). Helberger et al. (2017) argues that if we define vulnerability as the “limited ability to deal with commercial practices” (Duivenvoorde, 2013), then most digital marketing strategies, and if they are based on IoT data analysis, opaque algorithms, and sophisticated capabilities to influence behaviour and perception, have the capacity to turn the normally “average” consumer into a vulnerable one. This is particularly the case when connected services involve physical actuators under the skin or technologies that get under our skin in the sense of influencing the way we perceive ourselves and the world around us.

Moreover, the choice of factors for vulnerability appears quite arbitrary (Incardona & Poncibò, 2007). For consumers of connected cars, for example, the practice of using in-car cameras to determine when the user is hungry so that targeted advertising for outlets in the vicinity might be delivered through the infotainment system does not depend on inherent characteristics which make them as a group more vulnerable but dependencies common to us all. The consumer is caught in a situation in which she is vulnerable to that specific suggestion and the company has the power to detect and exploit that vulnerability. There is therefore a general or universal vulnerability which is converted through emotional and cognitive targeting based on IoT data sets combined with existing consumer profiles. This context renders people vulnerable. Hacker (2023) concludes from this that companies have the capability to change the type and effects of manipulative interventions based on individual profiles. Of course, the efficacy of these marketing strategies may be overblown, and the increase in consumer awareness of personalization in advertising can reduce their influence significantly. Even if this is the case, cyborg technologies can increase vulnerabilities to predatory business practices in other ways, for example, in subscription contracts or in private enforcement or upselling.

A restrictive interpretation of the UCPD case law is that inherent characteristics must be identified, and vulnerability cannot be relational or contextually sensitive unless it can be argued to be caused by credulity and applied to a clearly identifiable group. This means that personalization of IoT devices and services may create a way to sidestep the regulations for companies also—they target individuals based on individual profiles rather than groups (although the algorithms undoubtedly categorize people this is increasingly granular and obscure). One potential route for the protection of consumers rendered vulnerable due to personalized cyborgification may be found in the Enforcement and Modernisation Directive (EU) 2019/2161. This legislation provides for a consumer right of redress. So, when a consumer is unfairly targeted, made, vulnerable, and exploited, they have the right to individual remedies (such as ending the contract, obtaining a price reduction, or financial compensation). Compliance will involve various updates to domestic law (to be implemented by 2022). Although the Modernisation Directive will not be directly implemented by the UK post-Brexit, the Consumer Protection from Unfair Trading Regulations 2008 (which form the UK implementation of the UCPD) was amended (Sect. 4A) providing consumers a right to redress.

It is yet to be seen whether the threat of consumers undertaking such cases will have any deterrent effect. It is also worth noting that the Digital Markets, Competition and Consumers Bill, once adopted, will re-enact the provisions currently in the 2008 Regulations and will, crucially, extend the concept of vulnerability to include “the circumstances consumers are in” (s.245 4(d)). As it was introduced without much consideration or anticipation of the consequences of extending the concept to include significantly more contextual vulnerabilities, it will be interesting to see how this is implemented and interpreted in law and practice.

Inherent vulnerabilities are much more easily attributed to a group than variable relational and contextual factors which must be demonstrable and argued and so courts often are bound to an approach which foregrounds inherent vulnerabilities (Domurath 2018; Fineman, 2008; Friant-Perrot, 2016; Havrilla, 2017; Micklitz, 2018). However, credulity under the UCPD has the scope to be interpreted in a broader and more contextually sensitive way that provides greater protections for cyborg consumers. The guidance on the interpretation of the directive specifically states that it includes “context-dependent vulnerabilities” and that “[m]ulti-dimensional forms of vulnerability… are particularly acute in the digital environment.”

The CJEU has discussed the UCPD in various cases and credulity appears to refer to the use of false or misleading information that results in deception that leads to a transactional decision that the consumer would not have taken otherwise (see, for example, Joined Cases C-261/07 and C-299/07 VTB-VAB NV v Total Belgium NV and Galatea BVBA v Sanoma Magazines Belgium NV [2009] ECR I-02949; Decision Vj-5/2011/73 by the Hungarian Competition Authority, 10 November 2011). The Market Court of Helsinki has applied the concept of credulity in a case involving marketing materials seen as misleading as they took advantage of the credulity of consumers who were concerned about the environment (MAO: 157/11, the Market Court of Helsinki, 8 April 2011; European Commission, 2022a, 2022b). However, this concept has not been the subject of extended discussion, and contextualized vulnerabilities inherent in IoT consumer transactions have rarely been cited by the CJEU as a justification to protect consumers in the digital or IoT sectors. The idea of credulity in digital marketplaces has the potential to be interpreted in a way to expand protections to cyborg consumers more generally. It is, at present however, underdeveloped and does not take account of how the IoT and cyborgification increasingly give companies direct power over our bodies and physical environments. Restricting or banning certain types of business models that exploit these vulnerabilities in ways that might be construed as unfair commercial practices, based on analysis of how they identify or manufacture, cyborg vulnerabilities, may be a way of designing policy and law to better protect consumers. Alternatively, restricting access to influence and manipulation tools to types of (beneficent) actors, or for particular types of uses (as in the GDPR framework of purposes for data), may also be a potential path forward. Positive duties to design technology and IoT ecosystems more broadly in ways that promote “fairness by design” may also be worth consideration in consumer policy as well as reimagining the creation of a contract and consenting processes in relation to data processing. The Digital Services Act (DSA) Article 27, providing duties to design modification functionalities so consumers can understand and control the way that recommendation algorithms personalize services, is an example of this in action, and it will be interesting to see how this functions in relation to digital platforms and what lessons can be learned and translated into IoT technologies more generally.

### Unfair Contractual Terms

A two-tiered system distinguishing between consumers with inherent vulnerabilities and the rest is also applied in consumer law instruments such as the Unfair Contract Terms Directive (UCTD) where, although the directive only mentions “consumers,” the average consumer is the benchmark for assessing transparency of terms and conditions in consumer contracts in the CJEU (established by the CJEU in the Kásler case and recently applied in the A SA judgment).

Vulnerable consumers under the UCTD are assumed by the CJEU to also be protected when core terms are transparent for the average consumer. Esposito and Grochowski show that this may not be a sound assumption to make because transparency is a weak obligation and consumers do not generally have the resources to process the information, let alone act on it in a way that would be empowering (2022). In addition, the way that CJEU case law on the UCTD conceptualizes consumer vulnerability as arising primarily from informational asymmetries (which incidentally is often a contextual and relational factor rather than borne out of inherent deficiencies in the ability to process or retain information) means that vulnerability under the UCTD appears to consider much more than inherent characteristics (see, for example, CJEU decisions, such as Kásler, Bucura, Andriciuc, OTP Bank, GT, and, most recently, in A SA). Cyborg vulnerability is therefore arguably a relevant circumstance that judges should take into consideration when applying the fairness test in IoT-related cases.

This relational approach, foregrounding the asymmetries of power in a particular interaction, does not result in enhanced protections for vulnerable groups but a baseline level of transparency which is deemed to mitigate the asymmetry across the spectrum of consumer interactions (Riefa & Saintier, 2021). In the age of cyborg vulnerability where Things render people ever more susceptible to shaping of preferences and lifeworlds, personalized marketing, exploitative contracts, and extractive choice architectures, informational asymmetries are not enough to capture the way in which power is distributed and utilized to the detriment of consumers.

Transparency is clearly a pre-condition for consumer protection. But providing information prior to contractual agreements does nothing to protect consumers from being influenced or manipulated to agree to terms and conditions that are against their interests. This is particularly the case when IoT products create dependencies and services and companies give consumers the option to take it or leave it without the possibility of negotiation.

### Platform Regulation

While the understanding of vulnerability in UCPD or UCTD is rather plain, it seems more questionable in DSA, and other EU acts focused on the digital society and market which refer to groups of internet users that can be labelled as vulnerable in classical terms (such as minors), but at the same time, they address other types of market vulnerability—e.g., regarding (mis)use of consumer data—which can be framed as examples of cyborg vulnerability.

The Digital Services Act requires platforms to be more transparent and provides mechanisms by which they may be held accountable for their role in disseminating illegal and harmful content. Stricter obligations are imposed on very large online platforms and search engines (Sect. 5) which are required to offer users a system for recommending content that is not based on profiling (Article 38) and monitor the systemic risks of consumer harm they create (e.g., in relation to mental health) (Article 35).

Provisions such as Article 28 of the new DSA target vulnerable groups such as children for special protections and thus appear to adhere to the view that inherent vulnerabilities such as age provide a more persuasive political justification for enhanced consumer protections. Paragraph (81) of the introductory section of the Act, for example, refers to “the design of online interfaces which intentionally or unintentionally exploit the weaknesses and inexperience of minors.” An understanding of cyborg vulnerability might imply that such enhanced protections should not only apply to minors but should be more widely adopted.

Article 27 of the DSA specifically addresses the transparency of recommender systems used by online platforms. This provision requires platforms to clearly outline, in plain and intelligible language, the main parameters used in their recommender systems and any options available to users for modifying these parameters. The significance of this article in the context of cyborg vulnerabilities is multifaceted and underscores the need for greater user autonomy and protection in the digital environment. Moreover, Article 27’s requirement for platforms to monitor and report on the systemic risks of consumer harm, such as impacts on mental health, aligns with the broader goals of protecting against cyborg vulnerabilities and supporting their welfare as per Domurath (2018).

While provisions like Article 28 specifically target vulnerable groups such as minors, Article 27’s emphasis on transparency and user control has broader implications. Cyborg vulnerabilities, characterized by the intricate entanglement of technology with human identity and decision-making, suggest that such protections should extend beyond traditional vulnerable groups. Enhanced transparency and control mechanisms can benefit all users by safeguarding their autonomy and mitigating the potential for exploitation through digital interfaces.

### Defective Products

The vulnerability of the machine component in the human–machine hybrid is also vital to consider to be an aspect of cyborg vulnerability. The EU is putting greater emphasis on consumer vulnerability in relation to defective IoT products which, as a result, might be more vulnerable to cyberattacks or remote control by unwelcome actors. This is an aspect of what has been termed the “vulnerability of Things” which presents various risks for cyborg consumers. In the current version of the Product Liability Directive (PLD), products are defined as movables (Directive 85/374/EEC, Article 2). This is usually interpreted as excluding software although this is not explicit in the directive (Howells et al., 2017). This raises issues for cyborg consumer claims where software is not delivered in a movable object, like a USB stick, but can be downloaded, like an application (Written Question No. 706/88, 1989; Wuyts, 2014; Fairgrieve et al., 2016). A consumer installing a third-party software onto a device will therefore not necessarily be afforded protections if the software is defective and not fit for purpose under the old directive leaving them at risk of not having a legal claim for compensation in the event of damage. The suggested definition of products in the new Product Liability Directive proposals deals with the defectiveness of the abstract components of the modern-day cyborg (European Commission, 2022a, 2022b). Consumers are vulnerable to harms associated with defective products whether they fall into categories of vulnerable or average consumers. If Things are more vulnerable to defects or security breaches (due to their increased complexity, connectivity, network dependencies, and regular updates) and such defects are liable to creating damage of whatever kind, then cyborg consumers require additional legal protections. The question of what regulation is required varies from case to case. For example, autonomous cars (Livaak, 2018) and “smart” homes (Kenyon & Davies, 2019) where software defects could render users vulnerable to significant risks to life and limb may require more far-reaching product safety regulations as compared to smart watches that arguably carry less severe risks.

When products such as connected vehicles pose serious risks to life and limb, the regulation comes under the banner of product safety law rather than product liability law. The EU’s product safety regime is underpinned by the General Product Safety Directive and various sector-specific directives, while the product liability regime is governed by the Product Liability Directive. In addition, medical-grade wearables and autonomous vehicles are regulated specifically in the Medical Devices Regulation (MDR) (2017/745/EU) and the Vehicle General Safety Regulation (EU) 2019/2144. Together, these directives establish a comprehensive framework that combines proactive measures to ensure product safety and reactive measures to address harm caused by defective products.

Furthermore, the proposal introduces new factors to the non-exhaustive list considered by courts when assessing defective products and so considers some of the novel aspects of vulnerability introduced by cyborgification. Additions include interconnectedness, self-learning functions, and cybersecurity (EU Commission, 2022a, 2022b, Article 6.1). The scope of the concept of manufacturers will expand to include fulfilment service providers (Article 7 no. 3) and those that modify a product already placed on the market so long as the modifications are considered substantial and are undertaken outside the control of the original manufacturer (Article 7 no. 4). (2PLD Ch.2 (II)). This is intended to capture refurbished products, for instance.

Because of the complexity of the IoT ecosystem, where consumers use digital and hardware products and services provided and maintained by various organizations, these are a set of criteria that is difficult for consumers to establish. Because most products and services are provided in an interdependent ecosystem, where modification is routine, establishing that a defect was substantial and was conducted outside of the original manufacturers’ control appears to be an onerous and unnecessary hurdle to overcome in IoT cases.

In addition, because of the large variety of functions performed by IoT devices like smart watches or connected cars, even small modifications might have a disproportionate effect on consumers’ enjoyment of the product. It is therefore debatable how many actors will be able to practically argue the case for fulfiling this test. It appears that where universal concepts of vulnerability are the basis for intervention, the protections afforded to consumers tend to be weak, whereas inherently vulnerable populations are seen as candidates for much more muscular regulation. The argument against strong universal protections rests on the restrictions they may impose on innovation and market entry.

### Data Protection

Data protection laws in the EU, particularly the GDPR, are essential for safeguarding consumers within the IoT ecosystem. GDPR sets significant standards for the handling of personal data, with profound implications for consumer protection.

One key aspect of GDPR is the requirement for explicit and informed consent before processing personal data (Articles 6 and 7). In the IoT landscape, where data often fuels personalized services and advertising, companies must provide clear and transparent information regarding data collection. However, questions arise regarding GDPR’s effectiveness in preventing data from being used for undisclosed or manipulative purposes, given ambiguous purposes in privacy policies and lax enforcement.

GDPR also imposes limits on data collection, with data being strictly necessary for its intended purposes. Nevertheless, IoT companies often build extensive databases for personalization, resulting in inherently expansive data usage. Articles 13 and 14 of the GDPR mandate transparency in data processing by requiring data controllers to inform individuals about the existence, consequences, and logic of profiling activities. Additionally, Article 21 grants individuals the right to object to processing, including profiling for direct marketing, empowering them to control the use of their data. Article 22 addresses automated decision-making, including profiling, ensuring individuals have the right not to be subject to decisions based solely on automated processing which potentially has significant effects on individuals. Articles 5(1)(c) and 5(1)(b) emphasize the principles of data minimization and purpose limitation, respectively, which apply to profiling activities. Personal data processed for profiling should be adequate, relevant, and limited to what is necessary for the purposes for which it is processed.

Despite these regulations, it appears that IoT companies continue to operate relatively unhindered, often promoting personalization as a value proposition (more convenient and relevant “content” and advertising) rather than a threat to consumer privacy and autonomous decision-making capacity and thus soliciting consent from most consumers.

Consent represents one of the legal bases for processing personal data under GDPR, and IoT companies often rely on consent from individuals to process their data. Transparency is critical to ensure informed consent. However, the complexity of the technologies themselves, privacy policies, and consumer behaviour have raised concerns about the effectiveness of this strategy in adequately protecting consumers (Van Ooijen & Vrabec, 2019). Consumers tend to grant consent automatically when faced with a consent request (Custers et al., 2013). As IoT technologies proliferate, with smaller interfaces and algorithmic decision-making and actuation capabilities based on data, the obstacles to consumers maintaining autonomy over their data have clearly intensified (Cohen, 2019).

As data increasingly becomes a tangible concern for consumers, data protection laws and practices must adapt to address the very real risks to their interests, finances, safety, and security when entrusted to unscrupulous companies that can leverage data to make changes to a product remotely at any time.

The Digital Markets Act (DMA) references the GDPR and makes significant efforts to coordinate with it (see Recitals 12 and 37; Article 5(2)(2) DMA and Article 7(8) DMA). The Act aims to address some of the shortcomings observed in the GDPR’s enforcement, particularly concerning large tech companies like Facebook and Google, which have managed to circumvent many of the GDPR’s data collection limitations.

The DMA introduces stricter obligations for large digital platforms that control access to digital markets, to ensure fair competition and consumer protection by ensuring that gatekeepers provide clear and accessible information about data processing and obtaining genuine consent from users. It may be pointed out again that transparency measures often fail as a strategy to protect consumer interests in these markets (Jabłonowska & Tagiuri, 2023).

The inclusion of these measures in the DMA indicates a recognition of the need for a more robust approach to data protection in the digital age, especially concerning the pervasive influence of major tech companies. By aligning with the GDPR and addressing its enforcement gaps, the DMA aims to create a more comprehensive and effective regulatory environment that better safeguards consumer privacy and autonomy in the context of IoT and other digital technologies.

While the GDPR has established a critical foundation for data protection, its limitations in curbing the data practices of major tech companies highlight the need for additional regulatory measures. The DMA’s efforts to coordinate with the GDPR represent a significant step towards addressing these challenges and ensuring that consumers’ rights are more effectively protected in the evolving digital landscape.

### Artificial Intelligence

The proposed EU AI Act marks a groundbreaking effort to better regulate AI systems. It includes several laudable features, such as bans on widespread biometric surveillance, the use of AI systems to identify emotions, gender, or sexual orientation, as well as predictive policing systems. However, when it comes to safeguarding consumers’ rights within the IoT realm, there are several potential issues in the proposed legislative framework.

The AI Act proposals include Title II (Article 5) which bans certain high-risk uses of AI. Included in the suggested list are AI systems that deploy harmful manipulative “subliminal techniques” and AI systems that exploit specific vulnerable groups (physical or mental disability). This does not explicitly address the implications of algorithms on consumer privacy and data protection, nor do they explicitly link manipulation with unfair practices in IoT devices. The distinction between subliminal and other techniques has not been outlined, and it is difficult to see why unfair manipulation, of which consumers might be more consciously aware, should be permissible and how subliminality would be assessed. In addition, we can see the idea of inherent vulnerability already creating a dividing line to those susceptible to such manipulation and those for whom it is seen as sufficiently mentally capable of assessing the risks themselves.

It appears from EU briefing documents that many practices relating to AI usage in the consumer IoT may fall into the limited risk category which is described as systems that interact with humans (i.e., chatbots), emotion recognition systems, biometric categorization systems, and AI systems that generate or manipulate image, audio, or video content (i.e., deepfakes). These would only be subject to a limited set of transparency obligations. Given our arguments about cyborg vulnerability and the inadequacy of transparency obligations in achieving adequate consumer protections, this appears to provide myriad exceptions to the proposed ban on subliminal manipulation and could therefore fall short of what is needed.

The European Consumer Organisation (Oliveira da Silva, 2023) emphasizes that the proposed AI Act requires significant enhancements to ensure robust consumer protection. Oliveira da Silva (2023) argues that the proposal should adopt a broader scope by imposing fundamental principles and obligations, such as fairness, accountability, and transparency, upon all AI systems. This approach seeks to prevent potentially harmful practices more preventatively. In essence, BEUC advocates for an expansive framework that encompasses various aspects of AI deployment, going beyond the specific ban on subliminal techniques (Oliveira da Silva, 2023). Based on our analysis of cyborg vulnerability, we would support this position. This more universal approach to vulnerability would obviate the need to single out particularly vulnerable groups as requiring particular protections and thus excluding them from some of the potential benefits of personalized AI-based services.

### Implications for Consumer Law and Policy

In this article, we have interrogated the concept of cyborg vulnerability and applied it to the context of the consumer IoT. This has allowed us, to some extent, to shed new light on emerging legal and ethical concerns surrounding individuals whose bodies and identities are intertwined with these technologies. The concept of cyborg vulnerability can be utilized as a “machine of thought”—or a tool for critical analysis—in assessing the adequacy of various legal instruments.

The cyborg condition is established once the individual is immersed into the IoT ecosystem providing a constant stream of data, under surveillance, creating or consuming content created by other cyborg consumers and engaged by screens for considerable time each day. Over four billion people are members of this cyborg community. In practice, cyborgs are often treated by private companies as instruments within this system rather than as rights-bearing citizens (as “means” rather than “ends”), and consumer law from the UCPD, UCTD to the DSA, GDPR, and DMA has not yet succeeded in establishing more consumer-friendly practices.

As our reliance on integrated technological devices grows, so do the novel forms of consumer vulnerability. Cyborg vulnerability, though loosely defined in the past, has been developed and examined here, referring to the susceptibility of such individuals to data breaches, surveillance, unfair commercial practices, and unfair contractual terms.

Cyborgification means consumer expectations around privacy and security are changing. Our preferences and behaviours are shaping and being shaped by the IoT. In the world of constant surveillance and connection, our bodies and minds are reflected in us through data, and this shapes the stories that we tell about ourselves and the world around us. This may be seen more broadly, as an intervention on the perception of one’s own body and lifeworld. According to Noto La Diega (2022), in the “Internet of Bodies,” the online presence of bodies is changing the Internet, and, accordingly, the Internet is changing our bodies. In this paper, we argue that this extends even further to instigating changes in our identities and lifeworlds through the process of cyborgification. This creates novel benefits as well as risks in the form of cyborg vulnerabilities.

The transformation of the citizen-consumer into a cyborg plugged into the machine has significant implications for legal reform. When an individual enhances their physical and mental attributes using connected devices, on their person, in their home, and/or in their vehicle, they may make themselves more productive, more creative, or enhance some other capability. Despite these clear benefits, cyborgs are rendered highly dependent on hardware and software, and this makes them vulnerable to new forms of predatory business practices, cyberattacks, and novel types of damage (Ugo, 2022). The EU definition of vulnerable consumers should be expanded to consider universal and relational vulnerabilities created by cyborgification in the IoT. The structure of the UCPD and UCTD creating as it does a two-tier level of protection for average and vulnerable consumers should also be reconsidered. Universal protections, going beyond mere transparency measures and into regulating specific types of data gathering sharing and processing in relation to IoT products and services, should be considered and made fit for purpose in a world where business models and technologies can use data and remote control of devices to manufacture, target, and exploit cyborg vulnerabilities across the whole range of consumers. The test for unfairness may, rather than considering what would be fair for the fictional “average person” in a particular category of consumer (whether vulnerable or not), rather ask what fair practices in relation to this consumer in this set of circumstances would be and then ask whether restricting the corporate practice would not have a disproportionate effect on companies’ ability to conduct business.

From our discussion on the concept of relational vulnerability in the IoT, we can see that cyborg vulnerability can be exploited by IoT companies to cause harm. Greater transparency and reporting obligations, though not a solution, are a pre-requisite for smarter laws to be developed. Theorists advocate the relational approach’s view of obligations arising from cyborg vulnerability as extending beyond protection from harm to the provision of the social support necessary to promote autonomy (Nussbaum, 2006). It is this principle that underpins the positive duties of companies to protect the privacy and security of consumers by design contained in the General Data Protection Regulation (EU GDPR). Positive moral duties on companies to design their Things and business models in a way that avoids exploiting cyborgification follow from the Kantian principle of respect for persons. The law must be responsive to cyborg vulnerability, to the ways that our capacities for rational agency are rendered vulnerable by the architecture of the IoT environment and ecosystem. Relational, or even universal, approaches to cyborg vulnerability and regulators who are quick to identify and deal with novel threats to consumer interests might establish the conditions in which a more consumer-centric IoT might be brought to fruition.

Vulnerability in the age of the cyborg may be recategorized as a diverse and varied experience, but no less inevitable or widespread. Physical and mental vulnerabilities are enduring aspects of the human condition and cannot be legislated away but cyborg vulnerability may be more amenable to changes in law, policy, and practice which require those with the power to adapt the business models and technologies that construct the IoT and the digital environment to do so in ways that allow consumers greater autonomy. We can use the IoT as a beneficent tool of empowerment rather than a means of control and manipulation.

There appear to be inconsistencies in the approach, conceptualization, and implementation of these theories of vulnerability in consumer law, but a common thread is a universal vulnerability attributed to informational imbalances, and in other cases, there is a reversion to inherent vulnerabilities as the baseline for enhanced protections. A stronger protection prohibiting the intentional or unintentional exploitation of the weaknesses and inexperience of consumers generally may be required considering cyborgification.

In practice then, EU consumer law instruments and judgements target certain groups that are constructed as requiring protection due to inherent vulnerabilities, and the contextualized vulnerabilities in the IoT marketplace (i.e., digital and cyborg vulnerabilities) have been somewhat neglected. This conceptualization of vulnerability does of course depend both on the purpose of each instrument, and whether it is providing improved market conditions (necessitating an objective yardstick) or focusing on individual, subjective, instances of consumer harm. It appears that the DSA and other EU acts focused on the digital society and market like the draft AI Liability Act will function to protect some forms of digital and cyborg vulnerabilities, but it is arguably necessary to also update the UCPD and UCTD also to capture cyborg technologies that increase the power imbalance between producer and consumer to a degree as to create presumptions of universal vulnerability.

Restricting certain protections to the inherently vulnerable is understandable in some respects because of the risk of mission creep that an expansive concept of relational vulnerability might pose and the idea that this might stifle innovation. Given the way in which IoT markets are changing relations and interactions between producers and consumers, the argument that cyborgification (including processes such as personalization, digitalizations, servitization, and immersion in extractive choice architectures) renders us more universally vulnerable is gaining traction but as well as introducing industry-specific regulations in terms of AI and platforms, protection of cyborg consumers in the more general IoT market might be necessary.

Greater transparency is not enough to protect cyborg consumers. New rights of redress are a starting point, focusing on the outcomes of unfair practices rather than the fine points of a contractual agreement between the parties who are only theoretically informed about various terms and conditions. However, private enforcement may be a weak deterrent in practice because of the huge barriers for consumers to launch complaints or legal cases (i.e., the resources and time required). Collective enforcement is a necessity to avoid consumer protections becoming redundant. A lower threshold, in the form of a greater consideration of credulity and other contextual and relational vulnerabilities, for consumers to establish unfair practices would also be a promising development. This would better recognize the universal and relational characteristics of cyborg vulnerability.

Obligations on IoT companies to embed security and empowerment by design (in both the hardware and software) may also be required. There is also a compelling argument for the need to pause and reflect on whether the process of individual consumer contracts (the terms of which can now be personalized through AI and other technologies) is an adequate safeguard for consumer interests. Perhaps this process could be reimagined as a negotiation between smart AI assistants or a process of collective bargaining rather than a take-it-or-leave-it proposition. If this service becomes available, then providers of such services would have to be closely regulated also to prevent capture by vested interests.

Discussion on how cyborgification as a process could be understood theoretically through the lenses of governmentality (Foucault, 1980), performativity, dramaturgy (Goffman, 1961, 1969a, 1969b), habitus (Burdeau, 1989), and capability (Nussbaum, 2006, 2020) is required as well as empirical research (both statistical and generalizable, and contextual and detailed) to establish a strong evidence base upon which to design flexible and responsive regulatory systems of monitoring and enforcement.

## Data Availability

Data is available on request.
